# Challenges and opportunities in pediatric surgery training in Germany: a view from the trenches

**DOI:** 10.1186/s12909-025-06727-5

**Published:** 2025-02-04

**Authors:** Sabine Drossard, Louisa Schuffert

**Affiliations:** 1 Department of General, Visceral, Transplant, Vascular and Pediatric Surgery, University Hospital Würzburg, Oberdürrbacher Str. 6, 97080 Würzburg, Germany; 2 Department of Pediatric Surgery, University Hospital Mainz, Mainz, Germany

**Keywords:** Pediatric surgery, Surgical Education, Post-graduate training, Resident Training, Germany

## Abstract

**Introduction:**

Pediatric surgery training in Germany faces significant challenges related to structural issues and resource limitations, including variability in training sites and a lack of standardized oversight. This study aims to assess the current state of pediatric surgery training including its structure, quality, and resident satisfaction and identify areas for improvement.

**Materials and methods:**

We conducted an online survey between May 2022 and November 2023 using single-choice, multiple-choice and open-ended questions. Additionally, information was gathered via analysis of available statistics and through direct contact with the State Chambers of Physicians.

**Results:**

75 pediatric surgery residents and 15 young specialists participated in the survey. 12 of 17 state medical chambers responded to our inquiry, but only 4 maintain detailed statistics. Training often extends beyond the planned six years, mainly (75%) due to insufficient surgical exposure. Residents reported a predominant role of attendings in surgical training and other residents in clinical training. They desired more involvement from chiefs in their education. A significant proportion (58.9%) noted a lack of dedicated scientific education. Nearly half (44.4%) of the respondents had changed training sites, primarily due to clinical rotations (26,7%) and dissatisfaction with training conditions (30,0%).

**Conclusion:**

Pediatric surgery residents in Germany face inconsistent training quality and extended training periods, mainly due to insufficient surgical exposure. They report that some chiefs do not meet their obligations as trainers adequately. To address these issues, there is a need for enhanced oversight, standardized curricula, more surgical exposure and improved collaboration among training institutions.

**Supplementary Information:**

The online version contains supplementary material available at 10.1186/s12909-025-06727-5.

## Introduction

The present state of surgical training in Germany poses significant challenges for both future surgeons and their supervisors, mainly due to structural issues and resource limitations [1, 2]. Forthcoming political changes in the German healthcare system may significantly affect surgical training [1]. This is particularly true for pediatric surgery, as the field encompasses a broad spectrum of procedures and has limited training sites, primarily located in bigger hospitals. In Germany pediatric surgery covers not only general pediatric and newborn surgery but also many subspecialties including oncology, urology, traumatology, thoracic surgery, neurosurgery, and plastic surgery [3]. In 2023, there were 755 board certified pediatric surgeons working in Germany, 561 of them in hospitals [4]. No statistics are available regarding the total number and distribution of pediatric surgery trainees in Germany. According to a 2017 survey, there were 340 trainees across 77 hospitals, with an additional 21 trainees from 12 hospitals that did not participate in the survey [5]. Pediatric surgery training primarily takes place in hospitals. There is no centralized authority responsible for overseeing and standardizing resident training in Germany. Although the German Medical Association (*Bundesärztekammer*) provides a defined training curriculum (*Musterweiterbildungsordnung*), each State Chamber of Physicians (*Landesärztekammer*) has autonomy in adapting these guidelines into their specific program requirements. The curriculum outlines the minimum duration of training, mandatory and optional rotations as well as a list of diagnostic and surgical procedures that trainees must complete. The State Medical Chambers appoint instructors who are authorized to provide training according to the range of procedures carried out at their institutions. Consequently, not all hospitals can offer the complete training program, leading to variability in training standards across the country. This inconsistency makes it challenging to transfer between hospitals and states, which is often necessary to meet the required number of procedures a trainee must perform [6]. Pediatric surgery training in Germany encompasses 6 years, of which at least 48 months are dedicated to pediatric surgery, 6 months in an emergency department, and 6 months in a pediatric intensive care unit. There is an option to spend 12 months in another specialty [3]. There is only limited research on the structure and quality of pediatric surgery training in Germany. The most recent survey evaluating the training of pediatric surgery residents in Germany was published in 2010 [7]. This article aims to provide a comprehensive account of pediatric surgery training in Germany, providing statistical data and insights from residents’ perspectives with a focus on training structure, quality and trainee satisfaction.

## Materials and methods

All 17 State Chamber of Physicians were contacted via email in January 2024. Information was requested on the number of physicians currently in pediatric surgery training, the annual number of pediatric surgery board certifications awarded, and the number of authorized trainers. Additionally, the websites of the German Medical Association, the State Chambers of Physicians and their annual statistical reports were reviewed to obtain this information. Furthermore, an online survey was conducted among pediatric surgery residents between May 2022 and November 2023. The survey link was distributed via an existing email group of the resident working group within the German Society of Pediatric Surgery and during a yearly training course for pediatric surgery residents (*Akademie für Kinderchirurgie*, AKIC). The questionnaire was specifically developed for this survey based on literature research and included demographic items, questions about the duration and structure of training, as well as open-ended questions to gather subjective assessments of residents’ training experience, challenges faced, training preferences and other relevant topics. Responses were submitted anonymously to ensure that individual institutions could not be identified. The questionnaire consisted of 31 questions, including grouped questions, Likert scales, multiple-choice and single-choice questions, as well as open-ended questions (see supplementary material 1).

Residents were asked about their expectations and experiences on who provided their training in four aspects: (1) Clinical education: Learning how to take patients’ history, perform examinations, patient care on the ward and in the emergency room, carry out conservative therapy, practical skills such as wound care, IV insertion, etc. (2) Surgical education: Learning surgical steps and techniques. Assistance and guidance in the operating room. (3) Theoretical education: The specialist knowledge required to understand diseases, treatments, and surgeries—essentially textbook knowledge. (4) Scientific education: Learning to interpret scientific papers, conduct studies, and write scientific articles. Support with research projects, journal clubs, discussion of guidelines.

Responses were analyzed descriptively, with open-ended questions being categorized into clusters, which were established and reviewed by both authors. Statistical analysis of the data was conducted using Microsoft Excel 365 (Microsoft Corporation, Redmond, Washington, USA).

## Results

### Data from state chambers of physicians

**Fig. 1:**
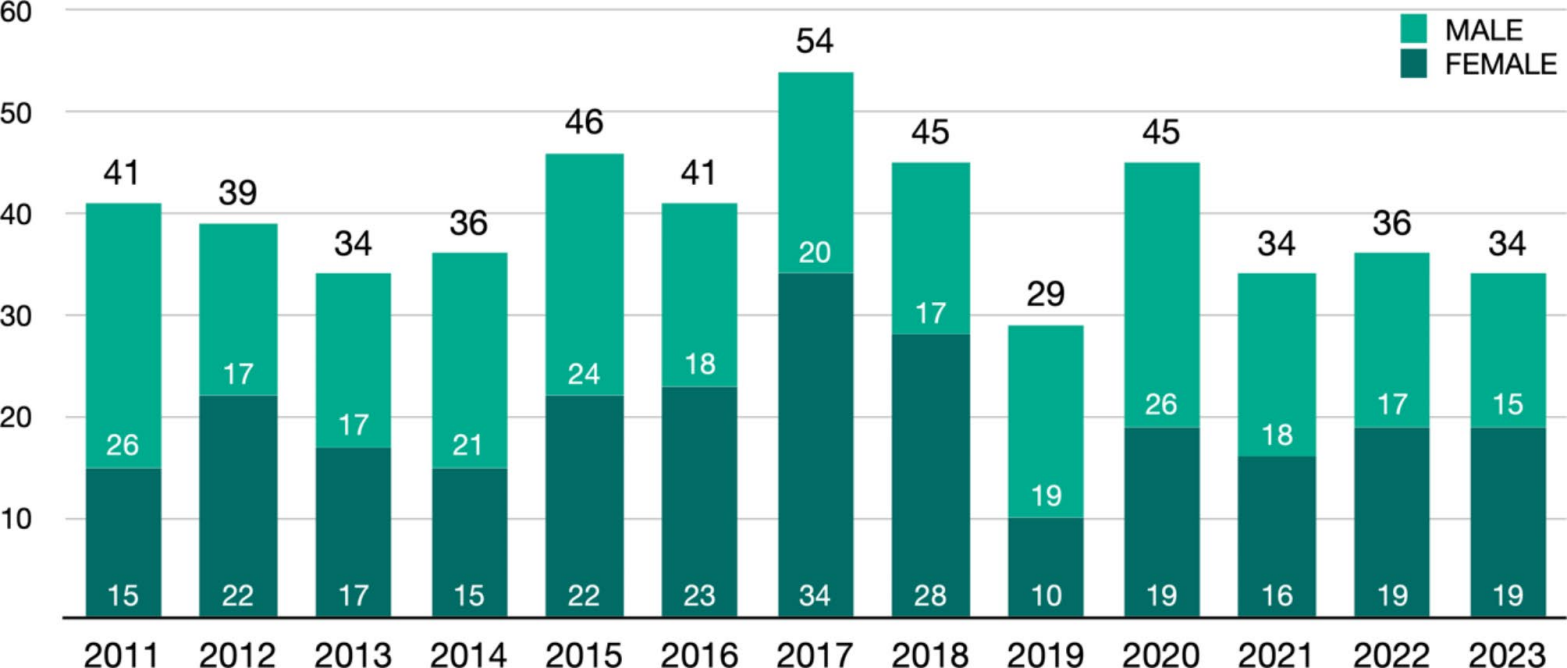
Number of board specializations per year. Source: Annual reports by the German Medical Association: Doctors’ statistic (*Ärztestatistik*, online via https://www.bundesaerztekammer.de/baek/ueber-uns/aerztestatistik)

Twelve State Chambers of Physicians responded to the email inquiry. Most provided annual reports via their websites, the German Medical Association also publishes annual statistical reports (*Ärztestatistik*). Four State Chambers of Physicians maintain statistics on pediatric surgery trainees. The remaining State Chambers of Physicians only interact with trainees at the time of registration for board examination. Notably, the Medical Association of Hesse surveys the number of physicians in training among authorized trainers across all specialties each year, but does not provide detailed information on pediatric surgery residents in their statistical report. Only the Medical Association of Westphalia-Lippe provided detailed statistics on the number of residents in pediatric residency: At the time of the inquiry, there were 45 individuals in pediatric surgery training, representing 0.62% of the total 7,308 residents in training. The duration of residents’ training at the time of their board examination is monitored only by the State Medical Chamber of Schleswig-Holstein. In this region the average training duration is 80 months, with approximately one board certification occurring per year. A total of 116 individuals are authorized to provide training in pediatric surgery in Germany. Over the last 13 years an average of 40 individuals per year have obtained board certification in pediatric surgery (Fig. 1) [4].

### Results of the online survey

**Table 1:** Demographics of participants

Year of training (*n* = 90)										
1	2	3	4	5	6	7	8	> 9		Specialist
5 (5,6%)	9 (10,0%)	16 (17,8%)	10 (11,1%)	10 (11,1%)	10 (11,1%)	11 (12,2%)	2 (2,2%)	2 (2,2%)		15 (16,7%)
**Training site (** ***n*** ** = 90)**										
University hospital with professorship in pediatric surgery									15 (16,7%)	
Department of pediatric surgery									40 (44,4%)	
Pediatric surgery as part of surgical department									10 (11,1%)	
Pediatric surgery as part of pediatric department									25 (27,8%)	
Private Practice									0 (0,0%)	
Other									2 (2,2%)	

75 pediatric surgery residents and 15 young specialists completed the survey. The demographics of participants are shown in Table 1. Only 2 of the 15 young specialists (13,3%) reported that they had finished their training in the scheduled timeframe of 6 years. Two thirds had been in training for more than 8 years (Table 2). Participants primarily cited difficulties in achieving the required minimum number of surgical procedures as the main reason for delayed completion of their training (stated by 75,0% of last-year residents and young specialists), followed by challenges in obtaining opportunities for mandatory clinical rotations (19,4%). 70% of participants indicated that they usually perform less than 3 surgeries per week, with 12,3% performing 0–1 and 28,8% performing 1–2 surgeries every week (Table 2). Reported reasons for non-fulfillment of the surgical catalogue are the total number and distribution of surgeries in relation to the number of residents, but also too much absent time because of shift work and the lack of more complex surgeries (Table 2).

**Table 2:** Training time and surgical exposure

**If you** ** are already a specialist: How long did your training take? (** ***n*** ** = 15)**												
6 years		6–7 years		7–8 years		8–9 years		9–10 years		> 10 years		
2 (13,3%)		2 (13,3%)		1 (6,7%)		8 (53,3%)		2 (13,3%)		0 (0,0%)		
**What are the reasons you are/were not able to finish your training within 6 years? (*n* = 36**,** residents in year 6 and above and specialists**,** multiple choice with additional free text**,** clustered)**												
Catalogue: Surgeries											27 (75,0%)	
Clinical Rotations: all											7 (19,4%)	
- *Clinical Rotation: Intensive care*											4 (11,1%)	
- *Clinical Rotation: Pediatrics*											1 (2,8%)	
- *Clinical Rotation: Emergency room*											1 (2,8%)	
- *Clinical Rotation: other departments / no full training license*											1 (2,8%)	
Pregnancy, parental leave, part time work											6 (16,7%)	
Change of specialty											3 (8,3%)	
Catalogue: Sonography, Central Lines, technical skills other than surgeries											2 (5,6%)	
Organizational reasons: waiting time for certificates, check of documents, exam and others											2 (5,6%)	
**If you did not fulfill the surgical catalogue**,** what are the reasons? (*n* = 90**,** multiple choice with additional free text**,** clustered)**												
Too many surgeries performed by specialists and attendings											56 (62,2%)	
Not enough surgeries performed by department in relation to number of residents											37 (41,1%)	
Too much absent time because of shift work											35 (38,9%)	
Too many basic surgeries, not enough complex ones											32 (35,5%)	
Surgeries performed too late in residency											20 (22,2%)	
Surgeries are not distributed evenly between residents											18 (20,0%)	
Too many complex surgeries, not enough basic ones											8 (8,9%)	
Absent time (parental leave, sick leave)											7 (7,7%)	
Other											6 (6,7%)	
**How many surgeries do you perform yourself per week? (*n* = 73**,** residents only)**												
0–1	1–2		2–3		3–5		5–7		8–10			> 10
9 (12,3%)	21 (28,8%)		21 (28,8%)		13 (17,8%)		7 (9,6%)		2 (2,7%)			0 (0,0%)

To address this problem, participants suggested the performance of partial steps during procedures, cooperation with other surgical departments and ambulatory settings, centralization of pediatric surgery, delegation of non-surgical tasks to other professions and particularly the implementation of standardized surgical curricula.

Almost half of the participants have changed their residency training site at least once. The most frequent reasons for changing are clinical rotations as part of the training (26,7%) and dissatisfaction with training, working conditions or their boss and team (30,0%). Only 6 participants changed their training site due to private reasons (Table 3).

**Table 3:** Changing of training sites

**Ho** **w many training sites have you worked at? (** ***n*** ** = 90)**					
1	2	3	4		5
50 (55,6%)	25 (27,8%)	10 (11,1%)	3 (3,3%)		2 (2,2%)
**Why did you change training site? (*n* = 40**, **multiple choice with additional free text**, **clustered)**					
Dissatisfaction				12 (30,0%)	
- *Dissatisfaction with training*				5 (11,1%)	
- *Dissatisfaction with working conditions*				3 (6,7%)	
- *Dissatisfaction with boss or team*				4 (8,9%)	
Organizational reasons				18 (45%)	
Rotation as part of the training (e.g. intensive care, pediatrics)				12 (26,7%)	
Temporary employment contracts				3 (6,7%)	
No full training license available				3 (6,7%)	
Career Optimization				6 (13,3%)	
Research possibilities				1 (2,2%)	
Career Improvement				2 (4,4%)	
Change to bigger hospital				2 (4,4%)	
Change to ambulatory setting				1 (2,2%)	
Other				14 (35,0%)	
Change of speciality				8 (17,8%)	
Private reasons				6 (13,3%)	

Participants rated their clinical and surgical skills in relation to their training time as sufficient, with an average score of 3.4 on a 5-point Likert scale (5 = very good, 1 = poor). Additionally, 58.9% of participants reported that their hospital does not provide a structured residency curriculum. Only 5.6% indicated the presence of a curriculum that is strictly followed, while another 18.9% reported partial adherence to the curriculum.

Participants indicated that most of their surgical education is delivered by attendings, while clinical education is primarily provided by fellow residents. They expressed a desire for greater involvement from their chiefs, particularly in theoretical training. Notably, 92.2% believe that their chief should be responsible for their theoretical education, compared to only 31.1% who currently experience this level of involvement. Furthermore, residents reported a lack of scientific education: 58,9% stated that no one at their training site is responsible for their scientific education (Table 4).

**Table 4:** Training responsibilities

Reality: Who is responsible for training you in this aspect at your training site? Expectation: Who do you think should be responsible? (*n* = 90, multiple choice)						
		Chief	Attendings	Specialists	Other Residents	No one
Clinical education	Expectation	55 (61,1%)	**85 (94**,**4%)**	57 (63,3%)	28 (31,1%)	0 (0,0%)
	Reality	13 (14,4%)	53 (58,9%)	34 (37,8%)	**65 (72**,**2%)**	11 (12,2%)
Surgical education	Expectation	71 (78,9%)	**88 (97**,**8%)**	43 (47,8%)	6 (6,7%)	0 (0,0%)
	Reality	40 (44,4%)	**84 (93**,**3%)**	30 (33,3%)	2 (2,2%)	4 (4,4%)
Theoretical education	Expectation	67 (74,4%)	**86 (95**,**6%)**	54 (60,0%)	32 (35,6%)	0 (0,0%)
	Reality	31 (34,4%)	**49 (54**,**4%)**	33 (36,7%)	34 (37,8%)	34 (37,8%)
Scientific education	Expectation	**83 (92**,**2%)**	70 (77,8%)	29 (32,2%)	12 (13,3%)	0 (0,0%)
	Reality	28 (31,1%)	20 (22,2%)	8 (8,9%)	10 (11,1%)	**53 (58**,**9%)**

Participants rated several aspects as highly important for their training, with the most critical being feedback after surgeries performed by residents, transparent allocation of surgeries to residents and briefing/debriefing of surgical procedures. However, residents rated the implementation of these aspects at their institutions poorly, with theoretical offsite training being the most valued aspect (Table 5).

**Table 5:** Importance and implementation of structured training

Expectation: How do you rate the importance of the following aspects for your training? Reality: To what extent are these aspects implemented/ valued at your training site? (Mean, 5-point Likert scale, 5 = very important, 1 = not important at all, *n* = 90)		
	Expectation	Reality
Feedback by attendings/colleagues after surgery	4,80	2,97
Transparent allocation of surgeries to residents	4,77	2,92
Briefing and debriefing of surgeries	4,73	2,63
Theoretical training (offsite)	4,70	3,59
Practical training (offsite)	4,66	3,13
Practical training (in-house)	4,59	2,14
Mentoring by senior colleagues	4,58	1,64
Structured residency curriculum	4,53	2,34
Feedback by chief in scheduled residency meetings	4,50	3,27
Theoretical training (in-house)	4,10	2,79
Rotations to other training sites	4,06	2,51

## Discussion

This study offers a comprehensive overview of pediatric surgery training in Germany, drawing on both statistical data and an online survey. By examining key aspects such as training structure, obstacles in meeting training requirements and resident satisfaction, it highlights the challenges and areas for improvement in the existing training programs. We identified a gap between the expectations of residents and the reality of training programs, highlighting its significance for the design of training structures and policy development. These findings are particularly relevant in the context of ongoing healthcare system reforms and the anticipated future demand for pediatric surgeons.

### Quality control and statistical analysis

The State Medical Chambers are responsible for specialist training in Germany. However, most chambers do not collect any data on the number of residents in training, time to complete specialization, breaks, quality of training sites, the existence of structured programs or other details. Out of the 17 medical chambers contacted, only 12 responded to our inquiry, and only 4 of these maintain relevant statistics. The only data available is the annual number of board examinations, which is published in the yearly Doctors’ Statistic by the German Medical Association. The Medical Chambers must fulfill their responsibility and closely monitor post-graduate surgical training to ensure high-quality surgical education. Statistical analyses and regular assessments of training quality are crucial for guiding necessary reforms in training programs.

### Number of trainees

Most surgical disciplines in Germany are experiencing a shortage of trainees and experienced specialists [1, 8–10], and pediatric surgery is now facing this issue as well [11]. There is no centralized strategy for the training of pediatric surgeons, requiring the pediatric surgery community to ensure that a sufficient number of specialists are trained to meet societal needs. Meanwhile, the German healthcare system is undergoing restructuring. While a broad range of basic medical treatments continue to be widely available, more specialized treatments are being centralized. Given the anticipated retirement of a significant number of pediatric surgeons in the coming years, it is challenging to project the necessary number of trainees required to ensure a high standard of pediatric surgery care.

### Duration of training

Although official statistics are lacking, the data presented strongly suggest that the majority of pediatric surgery trainees are unable to complete their training within the designated six-year period. If all pediatric surgery residents were to register for the board exam after 72 months of training, the annual average of 40 board exams would imply approximately 240 pediatric surgeons in training. However, with an estimated ~ 360 pediatric surgery trainees, the average training duration would extend to approximately 8 years and 9 months to account for the number of exams per year [11]. This estimation aligns with our survey data, where 53.3% of young specialists reported a training period of 8 to 9 years. Additionally, 23.3% of participants reported being in or beyond their sixth year of training and indicated that they would not complete their residency on time. The KarMed study, a longitudinal, multicenter cohort study of medical school graduates from seven German medical faculties, demonstrated that across all specialties, most trainees were able to complete their specialist training within the planned timeframe. However, the study did not differentiate between disciplines, leaving it unclear whether surgical trainees required extended training periods. Additionally, the study identified a correlation between longer training durations for female physicians and family formation, a pattern not observed in their male counterparts [12]. A study conducted by the Professional Association of German Surgeons (*Berufsverband der Deutschen Chirurgie*, BDC) in 2008 revealed that 45% of surveyed surgeons were unable to complete their training within the minimum required period [13]. Surgical training prompts residents to postpone starting a family [14], making prolonged training a significant disadvantage for female pediatric surgeons. With an increasing number of women entering the field [11], this issue poses a significant challenge for the future of pediatric surgery in Germany.

### Surgical exposure

Participants attributed their inability to complete training on time primarily to challenges in meeting the required minimum number of surgical procedures, followed by difficulties in securing opportunities for mandatory rotations. Trainees in pediatric surgery are required to perform a greater number of operations (410 according to the *Musterweiterbildungsordnung*) than those in other specialties, such as trauma surgery (375) or cardiac surgery (270) [3, 9]. Yet 40% of participating residents reported performing less than 2 surgeries per week. A lack of surgical exposure is considered problematic by surgical residents in different specialties [15–17], e.g. only 18% of cardiac surgery trainees reported being able to meet the required minimum number of cardiac surgeries by their sixth year of training [17]. But this issue is particularly significant in pediatric surgery due to the high volume and wide variety of procedures required. The heterogeneous structure of pediatric surgery in Germany, where the surgical spectrum varies considerably between hospitals, presents significant challenges for trainees and trainers. Therefore, every case should be regarded as a training opportunity, implementing the performance of partial surgical steps [1, 18]. Program directors claiming to offer comprehensive training in pediatric surgery must ensure access to a wide range of cases. Since case load is often beyond direct control, it may be necessary for trainees to rotate to other training sites to achieve a well-rounded education [1]. Furthermore, we expect that alleviating administrative burdens for residents through the implementation of digital tools, process optimization, and the delegation of non-surgical tasks to other healthcare professionals can improve surgical exposure and overall training efficiency.

### Changing of training sites

Nearly half of the participants in this study changed their training site at least once with clinical rotations accounting for 26.7% of the changes. The need to gain experience across multiple subspecialties often necessitates rotations to different institutions, a factor not unique to pediatric surgery. However, the fact that 30.0% of participants cited dissatisfaction with their training, working conditions, or supervisors as reasons for leaving their training site raises concerns about the consistency and quality of training environments. Studies in surgical education have shown that negative working environments, lack of mentorship, and inadequate support are key factors contributing to trainee burnout and attrition rates [19–22]. In a broader context, the KarMed study demonstrated that a supportive training environment plays a crucial role in the timely completion of specialist training [12]. Poor communication and strained relationships between residents and their supervisors can impede professional growth and lead to dissatisfaction, further delaying training progress [12, 20, 22]. Interestingly, only a small fraction of participants reported changing training sites for personal reasons, indicating that structural training aspects may be more important to residents than personal considerations. Strengthening collaboration among institutions, reducing bureaucratic barriers through medical chambers and standardizing the training regulations could ensure a more seamless transition for residents, minimize disruptions and enhance continuity of training. Implementing online teaching can also provide consistent education and support across different training sites, enhancing the overall training experience for pediatric surgery residents [23]. The high rate of site changes due to dissatisfaction suggests a need for improved oversight and standardization in training programs. Addressing issues such as inadequate supervision, inconsistent access to surgical opportunities and poor work-life balance could promote a more supportive training environment for pediatric surgery residents.

### Responsibility for training

Participants reported that surgical skills are predominantly taught by attendings, while clinical education largely comes from fellow residents. They expressed a strong preference for more involvement from their chiefs, particularly in theoretical training. Moreover, the lack of dedicated scientific education, with a majority of residents reporting no one responsible for this aspect at their training site, raises concerns about the emphasis on research and academic development in surgical training. These findings contrast with residents’ expectations and regulatory specifications, highlighting the need for a more structured approach to training and appropriate institutional supervision to ensure that chiefs fulfill their dedicated role in providing comprehensive surgical education.

### Limitations

Only basic demographic information was collected to maintain anonymity, limiting the performance of detailed subgroup analyses. Additionally, the reliance on self-reported data and relatively small number of participants may affect the generalizability of our findings. Despite these limitations, the survey provides the best available estimation in the absence of statistical data from the State Medical Chambers.

## Conclusion

This study highlights significant concerns in pediatric surgery training in Germany, particularly insufficient surgical exposure: residents frequently face delays in completing their training, largely due to difficulties in meeting the required number of surgical procedures. Departments seeking the privilege to train specialist pediatric surgeons should prioritize performing more cases as teaching cases to ensure trainees gain adequate exposure and experience. Furthermore, program directors need to ensure consistent access to surgical cases as well as structured theoretical and scientific education. It is crucial to standardize training programs and improve their supervision and statistical evaluation. The respective responsibility should be clearly defined and fulfilled. Hospitals need to reduce administrative and non-medical workload for residents by leveraging digital tools and delegating non-clinical tasks to allow more time for both theoretical and practical training. By fostering better institutional coordination and integrating digital education tools, we can support a more cohesive and effective training experience for pediatric surgery residents.

## Electronic supplementary material

Below is the link to the electronic supplementary material.

Supplementary Material 1

## Data Availability

The datasets used and analysed during the current study are available from the corresponding author on reasonable request.

## Declarations

### Author information

SD was the resident spokesperson and representative on the board of the German Society of Pediatric Surgery (Deutsche Gesellschaft für Kinderchirurgie) from 2019 to 2023 and deputy spokesperson from 2017 to 2019. LS was the deputy spokesperson from 2022 to 2023 and is the current resident spokesperson and representative on the board of the German Society for Pediatric Surgery.
