# Supplementary material for: Challenges and opportunities in pediatric surgery training in Germany: a view from the trenches
Source: BMC Med Educ. 2025 Feb 4;25:183. doi: 10.1186/s12909-025-06727-5 (PMC11796149; doi:10.1186/s12909-025-06727-5)
Supplement: Supplementary file 1 — Supplementary Material 1 [file 12909_2025_6727_MOESM1_ESM.docx]

**Questionnaire: Pediatric Surgery Training in Germany**

(Translated from German by SD)

1. **In what year of residency are you?**

- 1
- 2
- 3
- 4
- 5
- 6
- 7
- 8
- 9
- 10
- > 10
- I am a specialist

1. **What is your current workplace?**

- University hospital with professorship in pediatric surgery
- Department of pediatric surgery
- Pediatric surgery as part of surgical department
- Pediatric surgery as part of pediatric department
- Private practice
- Other

1. **How many training sites have you worked at**?

- 1
- 2
- 3
- 4
- 5
- > 5

1. **Why did you change training site?**

- Open text

1. **How would you rate your clinical and surgical skills (in relation to the stage of your training/the duration of your medical practice)?**

- Very good
- Good
- Neutral
- Poor
- Very poor
- I don’t know / prefer not to answer

1. **Do you have any comments on that?**

- Open text

1. **If you are already a specialist, how long did you take to complete your training?**

- Not applicable / still in training
- Exactly 6 years
- 6 – 6,5 years
- 6,5 – 7 years
- 7 – 7,5 years
- 7,5 – 8 years
- 8 – 8,5 years
- 8,5 – 9 years
- 9 – 9,5 years
- 9,5 – 10 years
- > 10 years

1. **If you took longer than the minimum time of 6 years for your training, what was the main reason for this? If you have been in training for more than 6 years, what is missing for you to be able to register for the exam? (Multiple choice)**

- Catalogue: surgeries
- Catalogue: ultrasound, central lines, technical skills other than surgeries
- Clinical rotation: intensive care
- Clinical Rotation: pediatrics
- Clinical rotation: emergency room
- Clinical rotation: no full training license
- Other (please specify):

1. **If you did not fulfill the surgical catalogue, what are, in your opinion, the reasons? (Multiple choice)**

- Not enough surgeries performed by department in relation to number of residents
- Surgeries are not distributed evenly between residents
- Too many surgeries performed by specialists and attendings
- Too much absent time because of shift work
- Surgeries performed too late in residency
- Too many complex surgeries, not enough basic ones (e.g. many oncolocig surgeries, only few inguinal hernias)
- Too many basic surgeries, not enough complex ones (.e.g many circumcisions, only few intestinal surgeries)
- Absent time (parental leave, sick leave)
- Other (please specify):

1. **Do you have any comments on the surgical training? Do you have suggestions on how it could be improved?**

- Open text

1. **Do you think that having children as a doctor in training negatively affects surgical training? (Multiple choice)**

- **No**
- **Yes, always**
- **Yes, but only for female trainees**
- **Yes, but only for male trainees**
- **Yes, but only when taking parental leave**
- **Yes, but only when working part-time**
- **I don’t know**

1. **Do you have any comments on the impact of family on surgical training?**

- Open text

1. **On average, how many surgeries do you perform yourself per week?**

- **0-1**
- **1-2**
- **2-3**
- **3-5**
- **5-7**
- **8-10**
- **> 10**

1. **Please rate the following statement: I am adequately supported by my supervisor in my training.**

- Strongly agree
- Agree
- Undecided
- Disagree
- Strongly disagree

1. **Please explain your answer: What would you wish for from your supervisor to support your training? What is still missing?**

- Open text

1. **Does your training site have a structured residency curriculum?**

- Yes, and it is adhered to
- Yes, but it is only partially adhered to
- Yes, but it is not adhered to
- No
- I don’t know

1. **Who is primarily responsible for the clinical-practical training of the trainees at your clinic? (e.g. patient care on the ward and in the emergency room, practical skills such as wound care, IV insertion, etc.) (multiple choice)**

- Chief
- Attendings
- Specialists
- Other Residents
- No one

1. **Who is primarily responsible for the surgical training of the trainees at your clinic? (Assistance and guidance in the operating room) (multiple choice)**

- Chief
- Attendings
- Specialists
- Other Residents
- No one

1. **Who is primarily responsible for the theoretical training of the trainees at your clinic? (Conveying specialist knowledge) (multiple choice)**

- Chief
- Attendings
- Specialists
- Other Residents
- No one

1. **Who is primarily responsible for the scientific training of the trainees at your clinic? (Support with research projects, journal clubs, discussion of guidelines, etc.) (multiple choice)**

- Chief
- Attendings
- Specialists
- Other Residents
- No one

1. **Who do you think should be primarily responsible for the clinical training of the trainees? (multiple choice)**

- Chief
- Attendings
- Specialists
- Other Residents
- No one

1. **Who do you think should be primarily responsible for the surgical training of the trainees? (multiple choice)**

- Chief
- Attendings
- Specialists
- Other Residents
- No one

1. **Who do you think should be primarily responsible for the theoretical training of the trainees? (multiple choice)**

- Chief
- Attendings
- Specialists
- Other Residents
- No one

1. **Who do you think should be primarily responsible for the scientific training of the trainees? (multiple choice)**

- Chief
- Attendings
- Specialists
- Other Residents
- No one

1. **Do you have any comments?**

- Open text

1. **Please rate the following aspects based on how important they are, in your opinion, for your training:**

|  | Very important | Important | undecided | Not very important | Not important at all |
| --- | --- | --- | --- | --- | --- |
| Structured residency curriculum | **O** | **O** | **O** | **O** | **O** |
| Rotations to other training sites | **O** | **O** | **O** | **O** | **O** |
| Transparent allocation of surgeries to residents | **O** | **O** | **O** | **O** | **O** |
| Theoretical training (in-house) | **O** | **O** | **O** | **O** | **O** |
| Practical training (in-house) | **O** | **O** | **O** | **O** | **O** |
| Theoretical training (offsite) | **O** | **O** | **O** | **O** | **O** |
| Practical training (offsite) | **O** | **O** | **O** | **O** | **O** |
| Mentoring by senior colleagues | **O** | **O** | **O** | **O** | **O** |
| Feedback by chief in scheduled residency meetings | **O** | **O** | **O** | **O** | **O** |
| Feedback by attendings/colleagues on clinical work | **O** | **O** | **O** | **O** | **O** |
| Feedback by attendings/colleagues after surgery | **O** | **O** | **O** | **O** | **O** |
| Briefing and debriefing of surgeries | **O** | **O** | **O** | **O** | **O** |

1. **Please rate the following aspects based on how important they are at your training institution / how well they are currently implemented: (Very important = completely implemented and supported, not important at all = not implemented at all)**

|  | Very important | Important | undecided | Not very important | Not important at all |
| --- | --- | --- | --- | --- | --- |
| Structured residency curriculum | **O** | **O** | **O** | **O** | **O** |
| Rotations to other training sites | **O** | **O** | **O** | **O** | **O** |
| Transparent allocation of surgeries to residents | **O** | **O** | **O** | **O** | **O** |
| Theoretical training (in-house) | **O** | **O** | **O** | **O** | **O** |
| Practical training (in-house) | **O** | **O** | **O** | **O** | **O** |
| Theoretical training (offsite) | **O** | **O** | **O** | **O** | **O** |
| Practical training (offsite) | **O** | **O** | **O** | **O** | **O** |
| Mentoring by senior colleagues | **O** | **O** | **O** | **O** | **O** |
| Feedback by chief in scheduled residency meetings | **O** | **O** | **O** | **O** | **O** |
| Feedback by attendings/colleagues on clinical work | **O** | **O** | **O** | **O** | **O** |
| Feedback by attendings/colleagues after surgery | **O** | **O** | **O** | **O** | **O** |
| Briefing and debriefing of surgeries | **O** | **O** | **O** | **O** | **O** |

1. **Do you have any comments?**

- Open text

1. **What is going well at your clinic regarding surgical training? What should be maintained? What could other clinics possibly learn from it?**

- Open text

1. **What is not going well at your clinic? What needs to change? How could these changes be implemented? In hindsight, what was missing in your own training?**

- Open text

1. **Do you have suggestions/ideas on how pediatric surgery trainees can be further supported? What would you wish for yourself?**

- Open text
